# Placenta-Derived Fetal Specific mRNA Is More Readily Detectable in Maternal Plasma than in Whole Blood

**DOI:** 10.1371/journal.pone.0005858

**Published:** 2009-06-10

**Authors:** Macy M. S. Heung, Shengnan Jin, Nancy B. Y. Tsui, Chunming Ding, Tak Y. Leung, Tze K. Lau, Rossa W. K. Chiu, Y. M. Dennis Lo

**Affiliations:** 1 Centre for Research into Circulating Fetal Nucleic Acids, Li Ka Shing Institute of Health Sciences, The Chinese University of Hong Kong, Prince of Wales Hospital, Shatin, Hong Kong Special Administrative Region, China; 2 Stanley Ho Centre for Emerging Infectious Diseases, The Chinese University of Hong Kong, Prince of Wales Hospital, Shatin, Hong Kong Special Administrative Region, China; 3 Department of Chemical Pathology, The Chinese University of Hong Kong, Prince of Wales Hospital, Shatin, Hong Kong Special Administrative Region, China; 4 Department of Obstetrics and Gynaecology, The Chinese University of Hong Kong, Prince of Wales Hospital, Shatin, Hong Kong Special Administrative Region, China; Stanford University, United States of America

## Abstract

**Background:**

Placental mRNA was detected in maternal whole blood, raising the possibility of using maternal blood for noninvasive prenatal diagnosis. We investigated fetal mRNA detection in maternal whole blood and determined if it offered advantages over maternal plasma analysis.

**Methodology:**

The concentrations of placental expressed genes, *CSH1*, *KISS1*, *PLAC4* and *PLAC1* in plasma and whole blood from healthy pregnant and non-pregnant individuals were compared by real-time quantitative reverse-transcriptase polymerase chain reaction analysis. Their fetal specificity was investigated by comparing the transcript concentrations in pre- and post-delivery samples and through SNP genotyping by matrix-assisted laser-desorption and ionization time-of-flight mass spectrometry. The gene expression profiles of pregnant and non-pregnant whole blood were investigated by microarray analysis. Upregulated genes in pregnant whole blood were selected for further quantitative analysis.

**Principal Findings:**

The concentrations of the four transcripts were significantly higher in third trimester maternal whole blood than corresponding plasma without significant correlations. *KISS1*, *PLAC4* and *PLAC1* were detected in non-pregnant whole blood but not plasma. The transcripts remained detectable in some postpartum whole blood samples. The *PLAC4* mRNA in maternal plasma showed fetal genotype while that in corresponding whole blood indicated both fetal and maternal contributions. Microarray analysis revealed upregulation of genes involved in neutrophil functions in pregnant whole blood including *DEFA4*, *CEACAM8*, *OLFM4*, *ORM1*, *MMP8* and *MPO*. Though possibly pregnancy-related, they were not pregnancy-specific as suggested by the lack of post-delivery reduction in concentrations.

**Conclusions:**

Maternal plasma is preferred over maternal whole blood for placenta-derived fetal RNA detection. Most studied ‘placental’ mRNA molecules in maternal whole blood were of maternal origin and might be derived from processes such as ‘illegitimate transcription’.

## Introduction

The analysis of fetal nucleic acids in maternal plasma holds much promise for noninvasive prenatal diagnosis [1]–[3]. Many investigations conducted to date were based on the detection of Y chromosomal sequences in maternal plasma [4], thus, limiting the utility of such applications to pregnancies involving male fetuses only. Circulating fetal mRNA analysis, on the other hand, offers a means for noninvasive prenatal assessment that is applicable to pregnancies regardless of the fetal gender [5]–[7].

Through the detection of the mRNA of placental expressed hormones, namely *chorionic somatomammotropin hormone 1 (placental lactogen)* (*CSH1*, NM_001317) and *chorionic gonadotropin, beta polypeptide* (*CGB*, NM_000737), the placenta was shown to be a source for fetal mRNA release into maternal plasma [5]. This observation facilitated the development of a microarray-based strategy to systematically identify placental expressed mRNA markers that were detectable in maternal plasma [6]. Furthermore, aberrant concentrations of placental expressed mRNA species have been shown to be associated with pregnancy complications, such as the elevation of *corticotropin releasing hormone* (*CRH,* NM_000756) mRNA in maternal plasma of preeclamptic pregnancies [7], [8]. In 2007, Lo et al. demonstrated that fetal chromosomal aneuploidy, such as trisomy 21, can be detected non-invasively from maternal plasma analysis by RNA-single nucleotide polymorphism (RNA-SNP) allelic ratio determination [9]. These studies suggest that circulating placental mRNA detection offers much opportunity for the development of noninvasive prenatal diagnostic or assessment applications.

Besides maternal plasma, maternal whole blood has been reported to be another medium for fetal mRNA detection [10]–[14]. *CSH1*, *CGB* and *placenta-specific 4* (*PLAC4*, NM_182832) mRNA concentrations were reported to be much higher in maternal whole blood than plasma [12], [14]. Indeed, the total RNA content of whole blood is higher than plasma. Hence, in this study, we investigated the detection of fetal mRNA in maternal whole blood and determined if it offered advantages over maternal plasma analysis.

## Materials and Methods

### Ethics statement

This study was conducted according to the principles expressed in the Declaration of Helsinki. Ethics approval from the Joint Chinese University of Hong Kong-New Territories East Cluster Clinical Research Ethics Committee was obtained (CRE-2005.131). All patients provided written informed consent for the collection of samples and subsequent analysis.

### Study design

Using real-time quantitative reverse-transcriptase polymerase chain reaction (QRT-PCR), we first compared the concentrations of four previously studied placental expressed transcripts, *CSH1*, *KiSS-1 metastasis-suppressor* (*KISS1*, NM_002256), *placenta-specific 1* (*PLAC1,* NM_021796) [5] and *PLAC4* [9] in maternal plasma and whole blood. Our previous microarray study [6] showed that these transcripts had much higher expression in placental tissues than blood cells. The relative placental-specificity of these transcripts are also supported by data in the public database, SymAtlas v1.2.4, Genomics Institute of the Novartis Research Foundation (Figure S1, S2, S3) [15]. Figure S1, S2, S3 shows that those transcripts are predominantly expressed in placental tissues as compared with the many other human tissues assessed by gene expression microarrays. We then determined the pregnancy-specificity of *CSH1*, *KISS1*, *PLAC1* and *PLAC4* by assessing their disappearance in post-delivery blood samples and detectability in blood samples of non-pregnant females and males. We further confirmed the fetal specificity of *PLAC4* and *chorionic somatomammotropin hormone-like 1* (*CSHL1*, NM_022579) mRNA by studying their genotypes in maternal plasma and whole blood using the RNA-SNP approach by matrix-assisted laser-desorption and ionization time-of-flight mass spectrometry. *CSHL1* is one of the potentially pregnancy-specific placental expressed transcripts identified in our previous microarray study [6]. Lastly, to exploit if more pregnancy-related circulating mRNA markers could be developed for maternal whole blood analysis, we mined for candidates after performing gene expression microarray comparison of whole blood samples from pregnant and non-pregnant individuals.

### Subjects and sample collection

Third trimester healthy women with singleton uncomplicated pregnancies (gestational age range, 38–39 weeks) were recruited with written informed consent from the Prince of Wales Hospital, Hong Kong. 12 mL of peripheral blood was collected into EDTA tubes before and at 24 hours after delivery. Placental tissues were collected immediately after delivery. Age-matched healthy non-pregnant individuals were recruited from the community.

### General sample processing

Blood samples were processed within 6 hours. 300 µL whole blood was added into 900 µL Trizol LS reagent (Invitrogen). The remainder of the whole blood sample was processed as previously reported for plasma and buffy coat [5], [9], [16]. All the processed blood samples were stored at −80°C until RNA or DNA extraction. Placental tissue samples were handled as per previous studies [5], [9].

### RNA extraction

For every 1.8 mL of plasma-Trizol-LS mixture and 1.2 mL of whole blood-Trizol-LS mixture, 200 µL and 240 µL of chloroform were added, respectively, and centrifuged at 12,000 *g* for 15 min at 4°C. The aqueous phase collected was mixed with 0.54 volume of 100% ethanol. All mixture from a single sample was applied to a RNeasy minicolumn (Qiagen) and processed following the manufacturer's protocol. Total RNA was eluted with 48 µL of RNase-free water. Placental RNA was extracted as reported [5], [9]. DNase I (Invitrogen) treatment was performed to remove genomic DNA contamination.

### DNA extraction

Maternal buffy coat and placental tissue DNA was extracted as previously described [9].

### Genes Investigated In This Study

Human genes: *CSH1*, chorionic somatomammotropin hormone 1 (placental lactogen) (NM_001317); *KISS1*, KiSS-1 metastasis-suppressor (NM_002256); *PLAC1*, placenta-specific 1 (NM_021796); *PLAC4*, placenta-specific 4 (NM_182832); *CSHL1*, chorionic somatomammotropin hormone-like 1 (NM_022579); *DEFA4*, defensin, alpha 4, corticostatin (NM_001925); *CEACAM8*, carcinoembryonic antigen-related cell adhesion molecule 8 (NM_001816); *OLFM4*, olflactomedin 4 (NM_006418); *FLCN*, folliculin (NM_144606); *ORM1*, orosomucoid 1 (NM_000607); *MMP8*, matrix metallopeptidase 8 (neutrophil collagenase) (NM_002424); *MPO*, myeloperoxidase (NM_000250).

### Real-Time QRT-PCR

All mRNA transcripts were quantified using one-step QRT-PCR. Calibration curves were prepared by serial dilutions of HPLC-purified single-stranded synthetic DNA oligonucleotides (Proligos, Singapore) with the amplicon specified. The sequence information of the primers, probes and calibrators as well as the reaction conditions are summarized in Table S1. QRT-PCR was set up as previously described [6], [9].

### RNA-SNP genotyping

Genotyping of *PLAC4* SNP, rs8130833, and *CSHL1* SNP, rs2246207, was performed involving steps of reverse transcription of RNA, PCR amplification, base extension and mass spectrometric analysis of the extension products [9]. DNA was extracted from placental tissues and maternal buffy coat for determination of the fetal and maternal genomic genotypes. The circulating *PLAC4* and *CSHL1* mRNA genotypes were then determined in maternal plasma and whole blood samples and further compared with the fetal and maternal genomic genotypes to confirm if they indeed originated from the fetus.

Protocols for the *PLAC4* SNP genotyping assay have been described previously [9]. Procedures for *CSHL1* were the same as *PLAC4* except that 454 ng placental RNA, 2.5 ng whole blood RNA or 48 µL plasma RNA was reverse transcribed at 60°C for 60 min followed by 85°C for 5 min. Placental cDNA of 2.5 µL was added to a total PCR volume of 25 µL. PCR reagents were added to 100 µL for plasma or whole blood cDNA. The PCR reaction mix was the same as that for *PLAC4* except 1× HotStar *Taq* PCR buffer was used for placental cDNA. The PCR was initiated at 95°C for 15 min, followed by 45 cycles of denaturation at 95°C for 20 s, annealing at 65°C for 30 s and extension at 72°C for 1 min with final incubation at 72°C for 3 min. 75 cycles were performed in the primer extension reaction for *CSHL1*. Primer sequences and molecular weights for the extension primer and extension products of each SNP allele are shown in Tables S2 and S3.

### Gene expression microarray analysis

From each of five third trimester pregnant women and five non-pregnant healthy females, 5 mL of peripheral blood was collected into PAXgene™ blood RNA tubes (PreAnalytiX) with further processing and RNA extraction according to manufacturer's instructions. DNase treatment was performed with RNase-Free DNase Set (Qiagen). 7 µg of extracted RNA from each sample was treated following the globin-reduction protocol recommended by Affymetrix [17]. The globin-reduced RNA was labeled and hybridized to the GeneChipH Human Genome U133A and U133B Arrays (Affymetrix, Santa Clara, CA) according to manufacturer's instructions. After hybridization, each array chip was washed, stained and scanned as reported [6]. The data were analyzed with GeneSpring v 7.2 (Agilent Technologies, Palo Alto, CA).

The microarray data in the. CEL format was imported and normalized using the following steps in sequence: (1) raw data processing by Robust Multi-chip Average, with GC-content background correction (GC-RMA); (2) data transformation whereby microarray data with values below 0.001 were set to 0.001; and (3) the signal intensity for each gene was divided by the median of its measurements in all samples. After filtering the gene-list with 70% confidence interval and non-parametric t-test analysis (P = 0.05), 2246 gene transcripts demonstrated differential expression between pregnant and non-pregnant blood samples. The gene list was further filtered by selecting transcripts with 1.5-fold higher expression in the pregnant compared with non-pregnant blood samples, and resulted in 247 gene transcripts. We sorted these genes in descending order of the fold difference.

### Statistics

Statistical analyses were performed using Sigma Stat (Systat).

## Results

### placental mRNA concentrations in third trimester maternal plasma and whole blood

Whole blood and plasma samples from ten third trimester pregnancies were analyzed. The median (range) concentrations for *CSH1*, *KISS1, PLAC4* and *PLAC1* mRNA in whole blood were 90 673 (9 324–387 010), 14 712 (0–71 544), 344 330 (88 940–552 686) and 12 498 (3 250–54 328) copies/mL, respectively. The median (range) concentrations for *CSH1, KISS1, PLAC4* and *PLAC1* mRNA in plasma were 11 338 (1 771–27 754), 535 (0–2 283), 4 317 (2 779–10 737) and 498 (0–1 276) copies/mL, respectively. The median concentrations of *CSH1, KISS1, PLAC4 and PLAC1* mRNA were 8-fold (P = 0.001, Mann-Whitney test), 27-fold (P = 0.017, Mann-Whitney test), 80-fold (P<0.001, Mann-Whitney test) and 25-fold (P<0.001, Mann-Whitney test) higher, respectively, in whole blood than plasma. Using the Spearman correlation test, no statistical significant correlation was observed between the whole blood and plasma mRNA signals for *CSH1* (R^2^ = 0.045, P = 0.583), *KISS1* (R^2^ = 0.003, P = 0.365), *PLAC4* (R^2^ = 0.013, P = 0.631), and *PLAC1* (R^2^ = 0.041, P = 0.631).

### Post-delivery clearance

**Figure 1:**
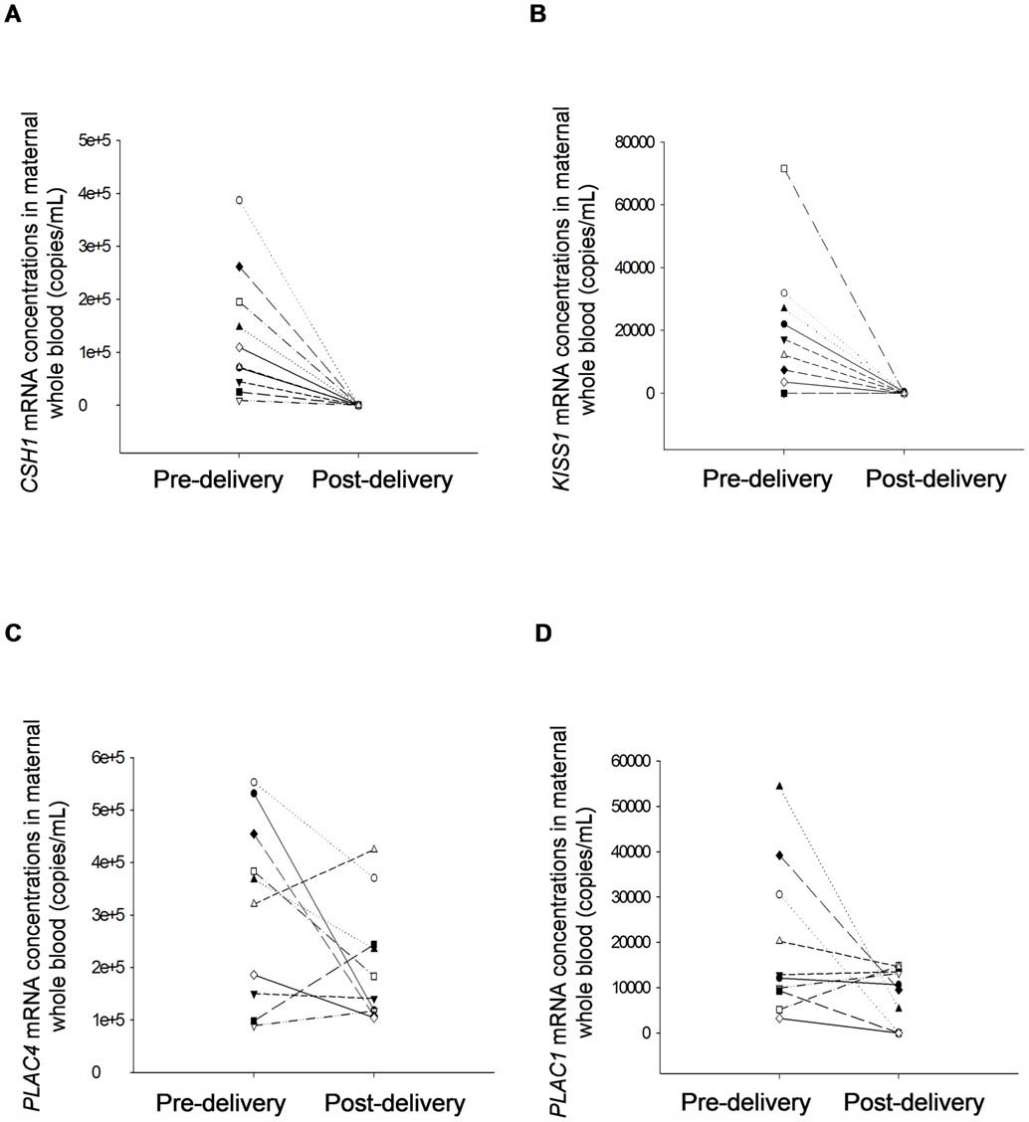
Concentrations of placenta-derived mRNA in pre- and 24-hour-post delivery maternal whole blood. Scatter plots of (A) *CSH1* (B) *KISS1* (C) *PLAC4* (D) *PLAC1* mRNA. Corresponding samples from each individual are connected by a line.

Whole blood and plasma samples were also collected 24 hours after delivery from the ten cases discussed above. The four transcripts were no longer detectable in all postpartum plasma samples. The plasma concentrations detected before and after delivery were statistically significant for all transcripts (P<0.001, Wilcoxon test). For the post-delivery whole blood samples, *CSH1* and *KISS1* mRNA became undetectable in 8 and 9 cases, respectively. However, *PLAC4* mRNA remained detectable in all cases at a median concentration of 162 203 copies/mL. *PLAC1* mRNA was detected in 7 post-delivery whole blood samples at a median of 10 081 copies/mL (Figure 1). Using the Wilcoxon test, the whole blood concentrations detected before and after delivery were shown to be significantly different for *CSH1* and *KISS1* (P<0.001) but not for *PLAC4* (P = 0.160) and *PLAC1* (P = 0.105).

### non-pregnant individuals

**Figure 2:**
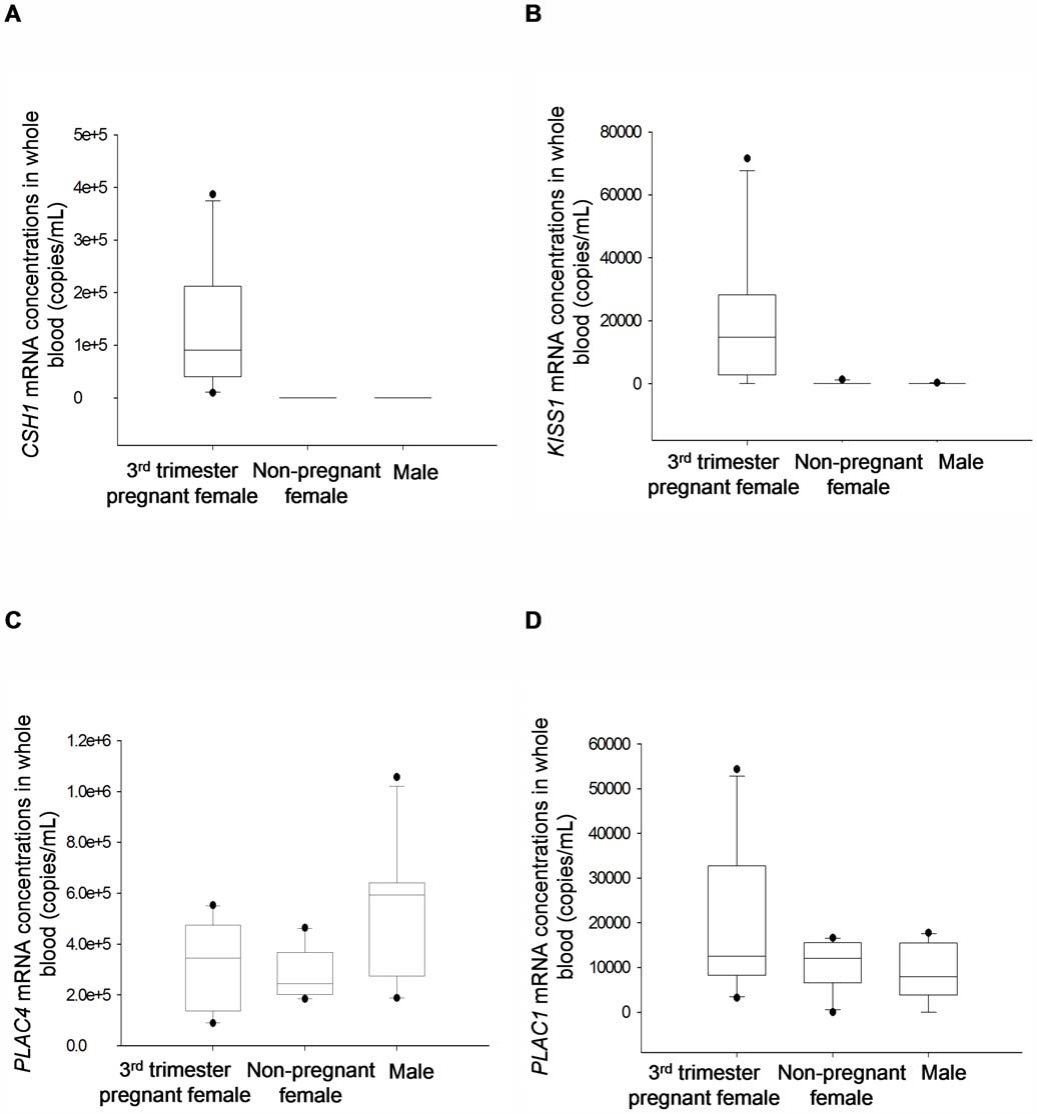
Concentrations of placenta-derived mRNA in third trimester pregnant and non-pregnant female and male whole blood. Box plots of (A) *CSH1* (B) *KISS1* (C) *PLAC4* (D) *PLAC1* mRNA. The line inside each box denotes the median. The lower and upper limits denote the 25th and 75th percentiles, respectively. The lower and upper whiskers denote the 10th and 90th percentiles, respectively. Filled circles denote the outliers. Asterisks denote all the data below detection limit of 8 copies per reaction.

*CSH1, KISS1, PLAC1* and *PLAC4* mRNA were not detectable in the plasma of 10 non-pregnant females and 10 males. Whole blood samples from these 20 individuals showed absence of *CSH1* mRNA. *KISS1* mRNA was detected in whole blood of 2 each of the non-pregnant females and males at 96 and 1 315 copies/mL in the former group and 264 and 300 copies/mL in the males. *PLAC4* mRNA was detected in all 20 whole blood samples with median concentrations of 244 498 copies/mL and 592 372 copies/mL, respectively, in non-pregnant females and males. *PLAC1* mRNA could be detected in whole blood of 9 non-pregnant females and 8 males at median concentrations of 12 024 copies/mL and 7 959 copies/mL, respectively. These concentrations were not significantly different from that of the third trimester whole blood samples for *PLAC4* (P = 0.065, Kruskal-Wallis one way analysis of variance (Kruskal-Wallis ANOVA) test) and *PLAC1* (P = 0.368, Kruskal-Wallis ANOVA test) (Figure 2).

### Genotyping of *PLAC4* and *CSHL1*

Third trimester placental tissues, maternal whole blood, buffy coat and plasma samples were collected. We aimed to compare the genotypes of the placental mRNA transcripts, *PLAC4* and *CSHL1*, in maternal plasma and whole blood with the genomic genotypes of the fetal-maternal pairs to confirm if the circulating transcripts were truly of fetal origin. *PLAC4* has a SNP, rs8130833, within the coding region [9]. *CSHL1* was targeted because it demonstrated high placental tissue expression at similar levels like *CSH1* which was highly homologous with other genes of the growth hormone cluster and that a target-specific RNA-SNP assay could not be developed [6].

The fetal and maternal genotypes for each pregnancy were first determined using placental tissue and maternal buffy coat DNA. Though nucleated fetal cells may be present at a density of 1 to 6 cells per milliliter of maternal blood in the samples [18], [19], such a level of “contaminant” would unlikely affect the predominant maternal genotype detected in maternal blood cells. Cases where the fetal and maternal genotypes differed were considered as informative and included 7 out of 28 cases for *PLAC4* and 11 out of 50 cases for *CSHL1*. The *PLAC4* and *CSHL1* mRNA genotypes were then determined in maternal plasma and whole blood RNA samples of the informative pregnancies. Representative mass spectra for *PLAC4* and *CSHL1* RNA-SNP genotyping are shown in Figures S4 and S5. The genotyping data of all informative cases are summarized in Table 1. For *PLAC4* mRNA, the genotype in maternal plasma corresponded to that of the fetus while the genotype in whole blood was always heterozygous indicating both fetal and maternal contributions. For *CSHL1* mRNA, the genotypes in both the plasma and whole blood samples were identical to the fetus.

**Table 1:** *PLAC4* and *CSHL1* RNA-SNP genotyping in third trimester maternal plasma and whole blood.

	Sample No.	DNA Genotype		RNA-SNP Genotype	
		Fetus	Mother	Plasma	Whole Blood
***PLAC4***	668	AG	AA	AG	AG
	696	AG	AA	AG	AG
	770	AG	AA	AG	AG
	774	AG	GG	AG	AG
	917	AG	AA	AG	AG
	1160	AA	AG	AA	AG
	1359	AA	AG	AA	AG
***CSHL1***	1172	CC	CT	CC	CC
	2432	CC	CT	CC	CC
	137	CT	CC	CT	CT
	140	CT	CC	CT	CT
	846	CT	CC	CT	CT
	847	CT	TT	CT	CT
	929	CT	CC	CT	CT
	2437	CT	TT	CT	CT
	2451	CT	TT	CT	CT
	2468	CT	TT	CT	CT
	2417	TT	CT	TT	TT

### Gene expression profile comparison between pregnant and non-pregnant female blood samples

The gene expression profiles of five third trimester and five non-pregnant female whole blood samples were compared using GeneSpring® v 7.2, (Agilent Technologies) software. The data discussed in this publication have been deposited in NCBI's Gene Expression Omnibus [20] and are accessible through GEO Series accession number GSE14771 (http://www.ncbi.nlm.nih.gov/geo/query/acc.cgi?token=hfavtosmyeoygzq&acc=GSE14771). The final gene list was generated by selecting transcripts with 1.5-fold higher expression in the pregnant than non-pregnant blood samples and resulted in 247 gene transcripts. We sorted these genes in descending order of the fold difference (Table 2). Twenty of the top 25 gene transcripts were found to be related to blood cell functions and more specifically the functions of neutrophils (13/25) (Table 2). However, placental expressed transcripts previously identified, namely *CSH1, CSHL1, KISS1, PLAC4* and *PLAC1* were not present on the list. *Defensin, alpha 4, corticostatin* (*DEFA4*, NM_001925), *carcinoembryonic antigen-related cell adhesion molecule 8* (*CEACAM8*, NM_001816), *olflactomedin 4* (*OLFM4*, NM_006418), *folliculin* (*FLCN*, NM_144606), *orosomucoid 1* (*ORM1*, NM_000607), *matrix metallopeptidase 8 (neutrophil collagenase)* (*MMP8*, NM_002424) and *myeloperoxidase* (*MPO*, NM_000250) which represented transcripts with large, medium and small differences in expression between pregnant and non-pregnant blood were further analyzed (Table 2).

**Table 2:** List of genes more highly expressed in whole blood of third trimester pregnant female than non-pregnant female.

aTranscripts	aGene Name	Probe Set ID	Genbank Accession No.	bFold Change	Expression in Pregnant Blood	Expression in Non-pregnant Blood
***defensin, alpha 4, corticostatin***	***DEFA4***	***207269_at***	***NM_001925***	***26.5***	***212.1***	***8.0***
***carcinoembryonic antigen-related cell adhesion molecule 8***	***CEACAM8***	***206676_at***	***M33326***	***22.9***	***373.9***	***16.3***
Transcribed sequences		231688_at	AW337833	21.4	206.2	9.6
lactotransferrin	LTF	202018_s_at	NM_002343	17.2	541.1	31.5
lipocalin 2	LCN2	212531_at	NM_005564	17.1	344.1	20.1
**olfactomedin 4**	***OLFM4***	***212768_s_at***	***AL390736***	***15.6***	***141.3***	***9.1***
defensin, alpha 1, myeloid-related sequence	DEFA1	205033_s_at	NM_004084	14.0	12060.0	864.5
cathelicidin antimicrobial peptide	CAMP	210244_at	U19970	13.7	531.9	38.8
cysteine-rich secretory protein 3	CRISP3	207802_at	NM_006061	12.2	80.7	6.6
***folliculin***	***FLCN***	***235250_at***	***AA992036***	***6.2***	***43.9***	***7.0***
chitinase 3-like 1	CHI3L1	209396_s_at	M80927	5.5	126.1	23.1
***orosomucoid 1***	***ORM1***	***205041_s_at***	***NM_000607***	***4.6***	***16.1***	***3.5***
polycythemia rubra vera 1	PRV1	219669_at	NM_020406	4.5	115.8	25.7
annexin A3	ANXA3	209369_at	M63310	3.8	275.8	73.0
POU domain, class 6, transcription factor 1	POU6F1	216332_at	L14482	3.7	39.2	10.5
transcobalamin I	TCN1	205513_at	NM_001062	3.7	87.9	23.8
carcinoembryonic antigen-related cell adhesion molecule 6	CEACAM6	211657_at	M18728	3.6	112.3	31.2
orosomucoid 2	ORM2	205040_at	NM_000607	3.6	67.9	19.1
thyroglobulin	TG	214977_at	AK023852	3.3	18.8	5.7
T-cell receptor interacting molecule	TRIM	217147_s_at	AJ240085	3.1	183.1	58.5
***matrix metalloproteinase 8***	***MMP8***	***207329_at***	***NM_002424***	***3.1***	***23.9***	***7.7***
S100 calcium binding protein A12	S100A12	205863_at	NM_005621	3.0	2963.0	982.4
hemogen	HEMGN	223669_at	AF130060	3.0	593.0	196.9
S100 calcium binding protein P	S100P	204351_at	NM_005980	3.0	1576.0	530.5
matrix metalloproteinase 9	MMP9	203936_s_at	NM_004994	2.8	428.2	155.7
⋮	⋮	⋮	⋮	⋮	⋮	⋮
neural proliferation, differentiation and control, 1	NPDC1	218086_at	NM_015392	1.5	29.9	19.6
motile sperm domain containing 2	MOSPD2	221895_at	AW469184	1.5	118.2	77.4
***myeloperoxidase***	***MPO***	***203949_at***	***NM_000250***	***1.5***	***8.5***	***5.6***
LOC401505	LOC401505	225036_at	BF969806	1.5	89.7	58.8

a Bold and italic fonts indicate the whole blood markers selected for QRT-PCR analysis.

b Fold change is the fold difference in expression level between the pregnant and non-pregnant blood samples according to the microarray data.

#### Quantification of the whole blood mRNA markers in third trimester whole blood and placental tissues

We assessed if the pregnancy-related whole blood mRNA transcripts were likely to have originated from the placenta. Placental tissue and whole blood samples were collected from ten pregnancies. The median mRNA concentrations for *DEFA4, CEACAM8, OLFM4, FLCN, ORM1, MMP8* and *MPO* were 0, 0, 3, 60, 0, 3 and 2 copies per nanogram placental RNA, respectively. Correspondingly, the median concentrations in whole blood samples were 125, 1181, 781, 207, 338, 386 and 200 copies per nanogram whole blood RNA in third trimester cases.

#### Concentrations of whole blood mRNA markers in pre- and post-delivery maternal whole blood

**Figure 3:**
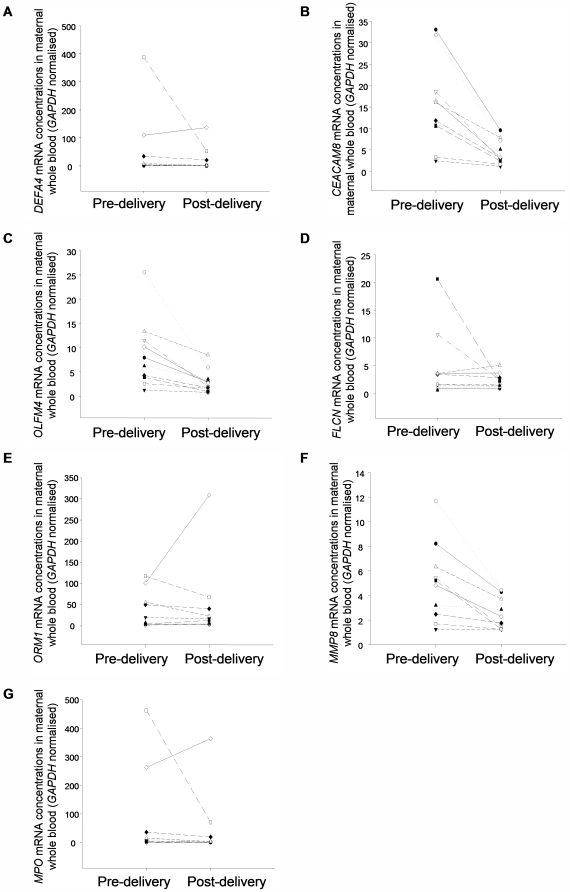
Quantitative analysis of identified whole blood mRNA markers in pre- and 24-hour-post-delivery maternal whole blood. (A) *DEFA4*. (B) *CEACAM8*. (C) *OLFM4*. (D) *FLCN*. (E) *ORM1*. (F) *MMP8*. (G) *MPO* mRNA. Corresponding samples from each individual are connected by a line.

After normalizing with *GAPDH* to adjust for the leukocyte concentration in whole blood, a statistically significant decreasing trend was observed in the post-delivery maternal blood samples for transcripts *DEFA4, CEACAM8, OLFM4* and *MMP8* comparing with the corresponding pre-delivery blood samples according to the Wilcoxon test (P<0.05). No significant difference was observed for *FLCN* (P = 1.00), *ORM1* (P = 0.63) and *MPO* (P = 0.06) (Figure 3).

#### Concentrations of whole blood mRNA markers in third trimester and non-pregnant whole blood

**Figure 4:**
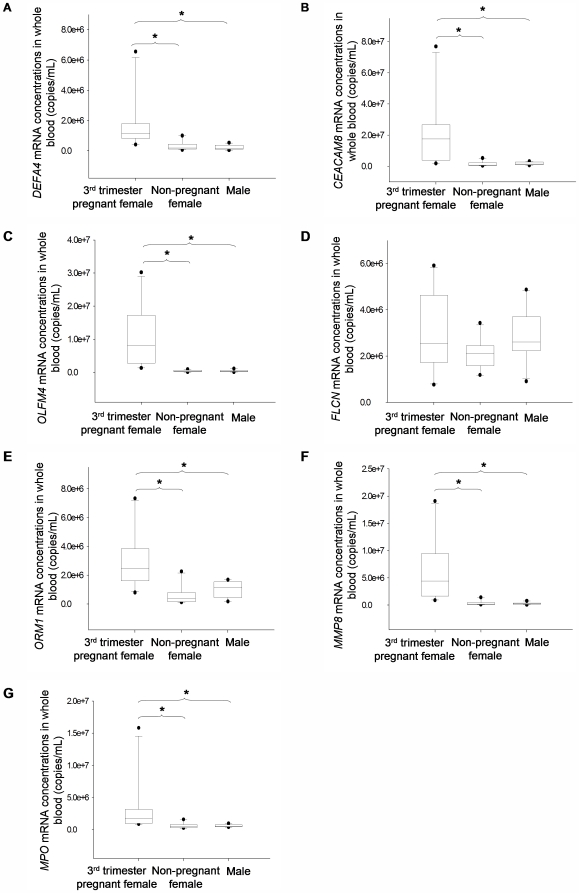
Concentrations of identified whole blood mRNA in third trimester pregnant and non-pregnant whole blood. Box plots of (A) *DEFA4* (B) *CEACAM8* (C) *OLFM4* (D) *FLCN* (E) *ORM1* (F) *MMP8* (G) *MPO* mRNA. The line inside each box denotes the median. The lower and upper limits denote the 25th and 75th percentiles, respectively. The lower and upper whiskers denote the 10th and 90th percentiles, respectively. Filled circles denote the outliers. Asterisks indicate the groups with significant differences in mRNA concentrations of the marker (P<0.05, Kruskal-Wallis test followed by pairwise comparison using the Student-Neuman-Keuls test).

When comparing the absolute concentrations of *DEFA4, CEACAM8, OLFM4, FLCN, ORM1, MMP8* and *MPO* mRNA in whole blood samples from third trimester pregnant, non-pregnant female and male, statistical significant differences were observed in the markers (P<0.001, Kruskal-Wallis ANOVA test) except *FLCN* (P = 0.231, Kruskal-Wallis ANOVA test) (Figure 4).

However, after normalization with *GAPDH* to adjust for the leukocyte concentration in whole blood (data not shown), significant differences among third trimester pregnant, non-pregnant female and male whole blood were only found for transcripts *OLFM4 and FLCN* (P<0.05, Kruskal-Wallis ANOVA test).

## Discussion

For decades, to develop noninvasive prenatal diagnosis, investigators have focused on the search for intact fetal cells in maternal blood [21], [22]. However, the rarity of such cells has hindered their robust identification in maternal blood [23]. The discoveries of cell-free fetal DNA [4] and later placental mRNA [5] in maternal plasma have offered new opportunities for the development of noninvasive prenatal diagnosis [9]. More recently, placental expressed and pregnancy-associated transcripts have been reported to be detectable in maternal whole blood [10]–[14]. As the RNA yield in whole blood far exceeds that of plasma, we explored in this study if maternal whole blood might represent a better specimen type for the detection of fetal specific placenta-derived mRNA.

We confirmed that placental expressed mRNA concentrations in third trimester whole blood were indeed higher than that in plasma for *CSH1, KISS1, PLAC4* and *PLAC1*. The observation was consistent with previous reports [12], [14]. While the four transcripts were not detectable in all post-delivery maternal plasma samples, there were no significant reductions in *PLAC4* and *PLAC1* mRNA concentrations in maternal whole blood samples collected 24 h after delivery. In non-pregnant females and males, all four placental transcripts were absent in plasma but *PLAC4* and *PLAC1* could be detected in the whole blood of both non-pregnant females and males. These data suggested that the *PLAC4* and *PLAC1* mRNA in whole blood could be derived from tissues or organs other than the placenta. This could also explain the lack of clearance of *PLAC4* and *PLAC1* mRNA from maternal whole blood after delivery. This is further supported by the lack of statistical significant correlations between the whole blood and plasma signals for *CSH1, KISS1, PLAC4* and *PLAC1* suggesting that the mRNA species detected in the two specimen types possibly have originated from different tissue sources.

It is believed that fetal or placental RNA is released into the maternal circulation due to apoptosis of the trophoblast cells [5], [24]. If our targeted RNA transcripts were indeed placenta-derived, they should carry the same genotype as the fetus. The concordance between the placental tissue and plasma RNA-SNP genotyping results for *PLAC4* and *CSHL1* confirmed the fetal origin of such mRNA molecules in maternal plasma. As for maternal whole blood mRNA, the *CSHL1* genotype was concordant with that of the fetus while the *PLAC4* genotype was always heterozygous even when the fetus was homozygous (cases 1160 and 1359 in Table 1). This latter observation suggests that there is maternal contribution to the detectable *PLAC4* mRNA in maternal whole blood. This finding confirms those reported by Go et al [25]. As can be appreciated from Table 1, pregnancies involving a homozygous fetus but a heterozygous mother (cases 1160 and 1359) are more useful for confirming the fetal specificity (i.e. absence of maternal transcript contamination) of the targeted transcript. The presence of a fetal-specific allele in maternal plasma (e.g. G-allele in case 668) merely suggests the existence of fetal contribution to the mRNA pool but does not indicate that there is no maternal transcript contamination. Yet, it is the absence of the maternal specific allele (e.g. G-allele in case 1160) that confirms the fetal-specificity of the transcript.

Concluding from our data, *CSH1*, *CSHL1* and *KISS1* appear to be pregnancy-specific in both plasma and whole blood. On the contrary, *PLAC4* and *PLAC1* are pregnancy-specific only in plasma but not whole blood which has both fetal and maternal contributions. Thus, it is not surprising that Banzola et al reported a lack of quantitative difference in whole blood *PLAC4* (located on chromosome 21) mRNA between euploid and trisomy 21 pregnancies [14]. Our data showed that there was no statistical significant difference in whole blood *PLAC4* mRNA concentrations even between pregnant and non-pregnant individuals. One possible explanation of the maternal contribution to these apparently “placenta-derived” transcripts in whole blood may be related to the phenomenon of illegitimate expression that lymphocytes had been shown to express transcripts irrelevant to their functions [26]–[28]. Microarray analyses do not reveal these expression profiles because microarray expression data are often presented in a relative scale where the extremely low abundance signals will be dwarfed by the high abundance signals. These low abundance signals in blood cells may become a significant contaminant in terms of absolute quantities when whole blood is the biological sample analyzed.

Nonetheless, *CSH1*, *CSHL1* and *KISS1* appeared to be pregnancy-specific in whole blood. Thus, we systematically investigated if other pregnancy-specific whole blood mRNA markers could be developed based on microarray comparison of pregnant and non-pregnant whole blood samples. We found 247 genes which showed at least 1.5-fold elevation in the former than the latter group. Thirteen of the top 25 transcripts on the list were related to blood cell functions, especially that of neutrophils. This is not surprising as pregnancy has been reported to induce inflammatory change in peripheral blood and neutrophils are major contributors to inflammation [29], [30]. Unexpectedly, none of the previously studied placenta-derived transcripts, *CSH1*, *KISS1*, *PLAC4* and *PLAC1*, appeared on the gene list. This was also the case as reported by Maron et al [13]. According to our QRT-PCR data on the pregnancy-related whole blood markers such as *DEFA4*, the median expression levels of these genes were much higher than that of the placenta-derived transcripts in whole blood (Figures 2 and 4). Therefore, it is possible that *CSH1*, *CSHL1* and *KISS1* were ranked much lower than 247. Furthermore, as *PLAC4* and *PLAC1* were readily detectable in both pregnant and non-pregnant whole blood and possibly without the 1.5-fold threshold difference in expression, they would not have appeared on the sorted gene list.

We have validated the pregnancy-specificity of 7 transcripts with differences in expression between pregnant and non-pregnant whole blood as determined by the microarray analysis. *DEFA4* is involved in host defense and found in neutrophils [31]. *CEACAM8* is expressed only in neutrophils and eosinophils in humans with undetermined function [32]. *OLFM4* is previously recognized as human granulocyte colony stimulating factor stimulated clone-1 with high expression in cancerous tissues [33]. *FLCN* is speculated to be a tumor suppressor gene with unclear function [34]. *ORM1* encodes a key acute phase plasma protein [35]. During pregnancy, *ORM1* is suggested to be involved in the maintenance of homeostasis between maternal and fetal systems within the chorioallantoic placenta [36]. *MMP8* is expressed in diverse cell types including neutrophils, macrophages, T cells, epithelial cells and endothelial cells in inflammatory conditions [37], [38]. *MMP8* was also detected in the chorion during labor and was proposed to be a predictive marker for preterm delivery [39], [40]. *MPO* is a heme protein primarily hosted in human polymorphonuclear neutrophils [41]. During pregnancy, *MPO* was observed on the surface of neutrophils [42].

Our QRT-PCR validation of the whole blood markers showed a statistically significant difference between the expression levels among third trimester pregnant, non-pregnant female and male individuals for *OLFM4* and *FLCN* only after normalization with the corresponding *GAPDH* data. All markers, except *FLCN*, were related to the hematopoietic system, especially the neutrophils. It has also been reported that the total leukocyte count would rise during pregnancy with neutrophils accounting for most of the increased leukocyte count [43], [44]. Hence, the increased expression level observed from the microarray data was probably due to the augmented leukocyte number instead of the transcript expression level in the cells. This may also explain why we found a significant difference in concentrations of those transcripts only when directly comparing the pregnant and non-pregnant whole blood samples but not after *GAPDH* normalization.

The whole blood microarray study identified transcripts whose functions were related to the physiological changes of pregnancy. However, their presence in non-pregnant whole blood and lack of clearance after pregnancy suggested that they were not “pregnancy-specific” markers, albeit being “pregnancy-related”. As they were also detectable in blood of non-pregnant individuals, they were therefore not fetal-specific either. Concluding from all the data in this study, it appears that placenta-derived fetal-specific transcripts can be more readily identified from maternal plasma than whole blood. While some transcripts are fetal-specific in maternal whole blood, e.g. *CSHL1*, due care is needed to validate each new potential whole blood transcript that is meant to be used as a circulating fetal RNA marker. Illegitimate expression by maternal blood cells would need to be excluded before adopting such a marker.

## Supporting Information

Figure S1 Bar-charts adopted from Human GeneAtlas GNF1H showing expression of (A) CSH1 (208356_x_at) and (B) CSHL1 (205958_x_at) in different human tissues. (http://symatlas.gnf.org/SymAtlas)
(2.28 MB TIF)

Figure S2 Bar-charts adopted from Human GeneAtlas GNF1H showing expression of KISS1 (205563_at) in different human tissues. (http://symatlas.gnf.org/SymAtlas)
(0.17 MB TIF)

Figure S3 Bar-charts adopted from Human GeneAtlas GNF1H showing expression of (A) PLAC4 (214750_at) and (B) PLAC1 (219702_at) in different human tissues. (http://symatlas.gnf.org/SymAtlas)
(2.60 MB TIF)

Figure S4 Mass spectra showing PLAC4 RNA-SNP genotypes of placenta, third trimester maternal plasma and whole blood samples. (A) a homozygous placental RNA sample showing a single peak A with unextended primers (UEP). (B) third trimester maternal plasma RNA showing a single peak A as the placental RNA sample. (C) third trimester maternal whole blood RNA showing a peak A and a minor peak G as indicated by the arrow.
(1.09 MB TIF)

Figure S5 Mass spectra showing CSHL1 RNA-SNP genotypes of placenta, third trimester maternal plasma and whole blood samples. (A) a heterozygous placental RNA sample showing 2 peaks, allele C and allele T with unextended primers (UEP). (B) third trimester maternal plasma RNA also showing 2 peaks as the placental RNA sample. (C) third trimester maternal whole blood RNA showing both peaks of allele C and allele T.
(1.07 MB TIF)

Table S1 Primer, probe and calibrator sequences and reaction conditions for quantitative real-time RT-PCR assays.
(0.08 MB DOC)

Table S2 Primer sequences for reverse transcription and PCR amplification of the PLAC4 and CSHL1 SNP.
(0.03 MB DOC)

Table S3 Sequences and molecular weights of the extension primer and the expected extension products for each of the alleles of the PLAC4 and CSHL1 SNPs.
(0.03 MB DOC)
