# Supplementary figures and images for: Placenta-Derived Fetal Specific mRNA Is More Readily Detectable in Maternal Plasma than in Whole Blood
Source: PLoS One. 2009 Jun 10;4(6):e5858. doi: 10.1371/journal.pone.0005858 (PMC2690655; doi:10.1371/journal.pone.0005858)

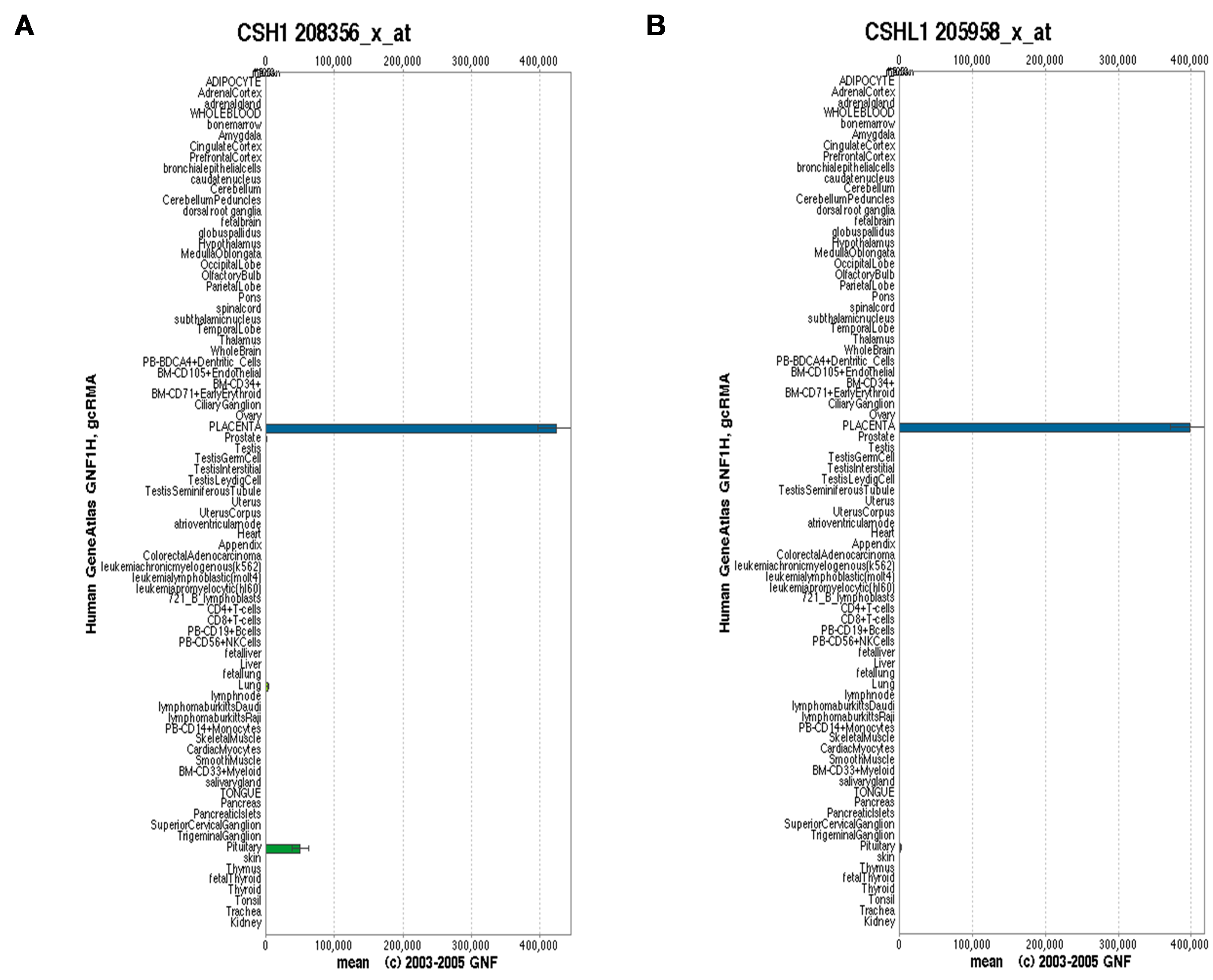

Supplement: Figure S1 — Bar-charts adopted from Human GeneAtlas GNF1H showing expression of (A) CSH1 (208356_x_at) and (B) CSHL1 (205958_x_at) in different human tissues. (http://symatlas.gnf.org/SymAtlas) (2.28 MB TIF) [file pone.0005858.s001.tif]

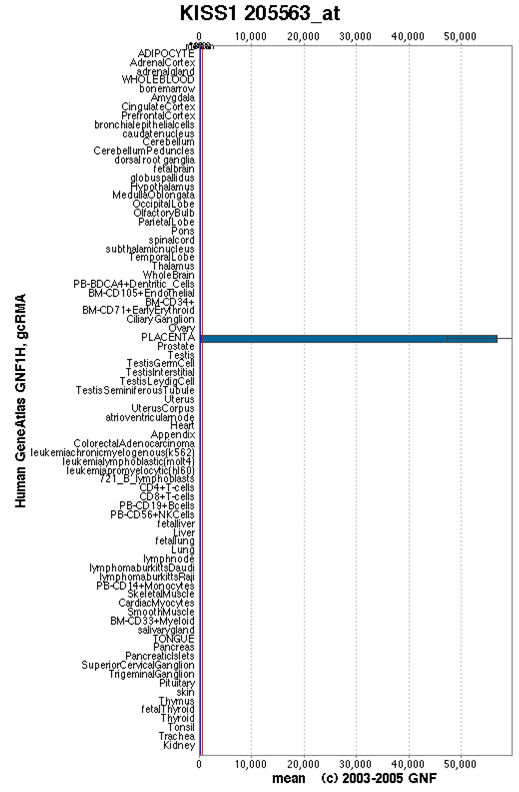

Supplement: Figure S2 — Bar-charts adopted from Human GeneAtlas GNF1H showing expression of KISS1 (205563_at) in different human tissues. (http://symatlas.gnf.org/SymAtlas) (0.17 MB TIF) [file pone.0005858.s002.tif]

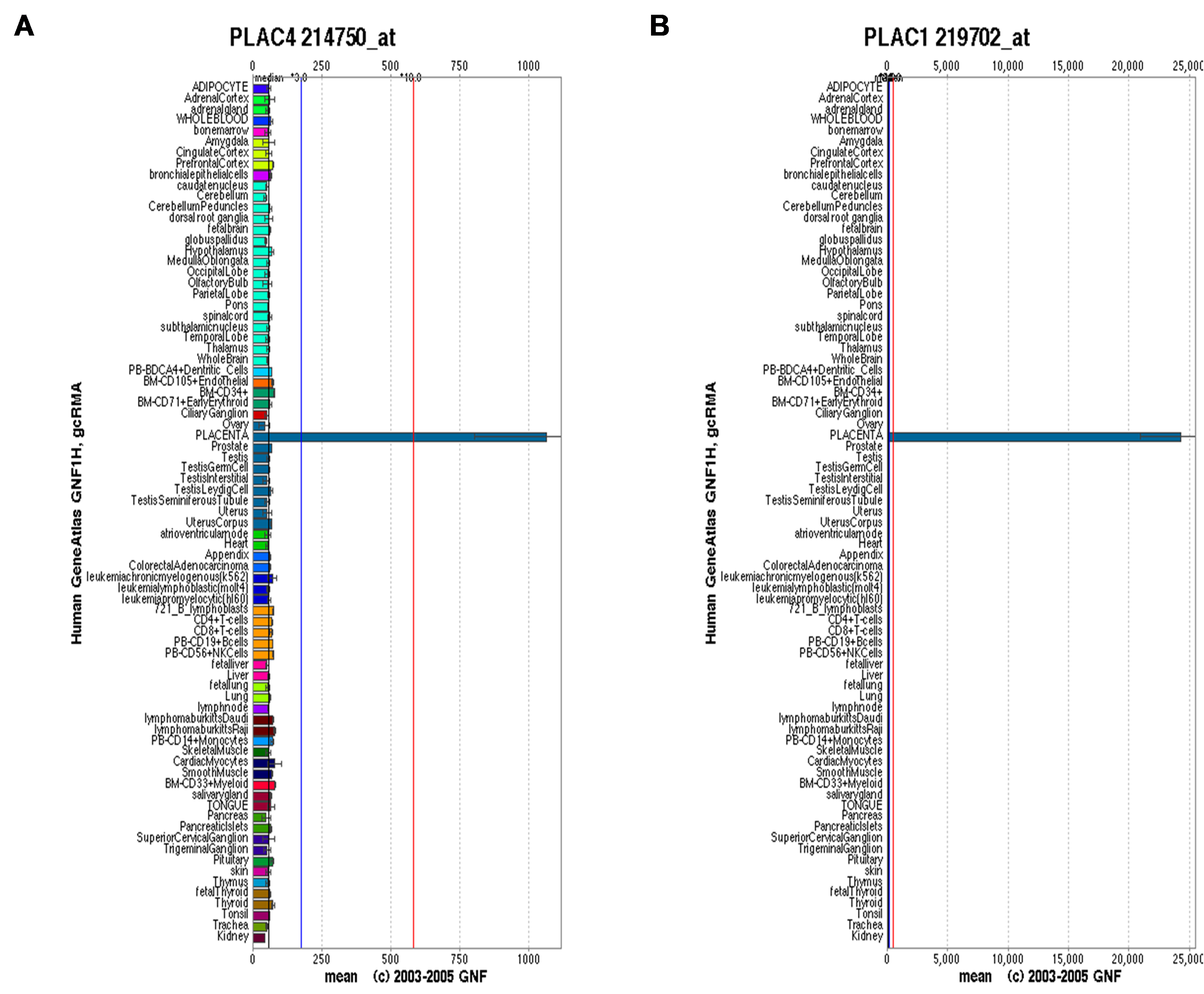

Supplement: Figure S3 — Bar-charts adopted from Human GeneAtlas GNF1H showing expression of (A) PLAC4 (214750_at) and (B) PLAC1 (219702_at) in different human tissues. (http://symatlas.gnf.org/SymAtlas) (2.60 MB TIF) [file pone.0005858.s003.tif]

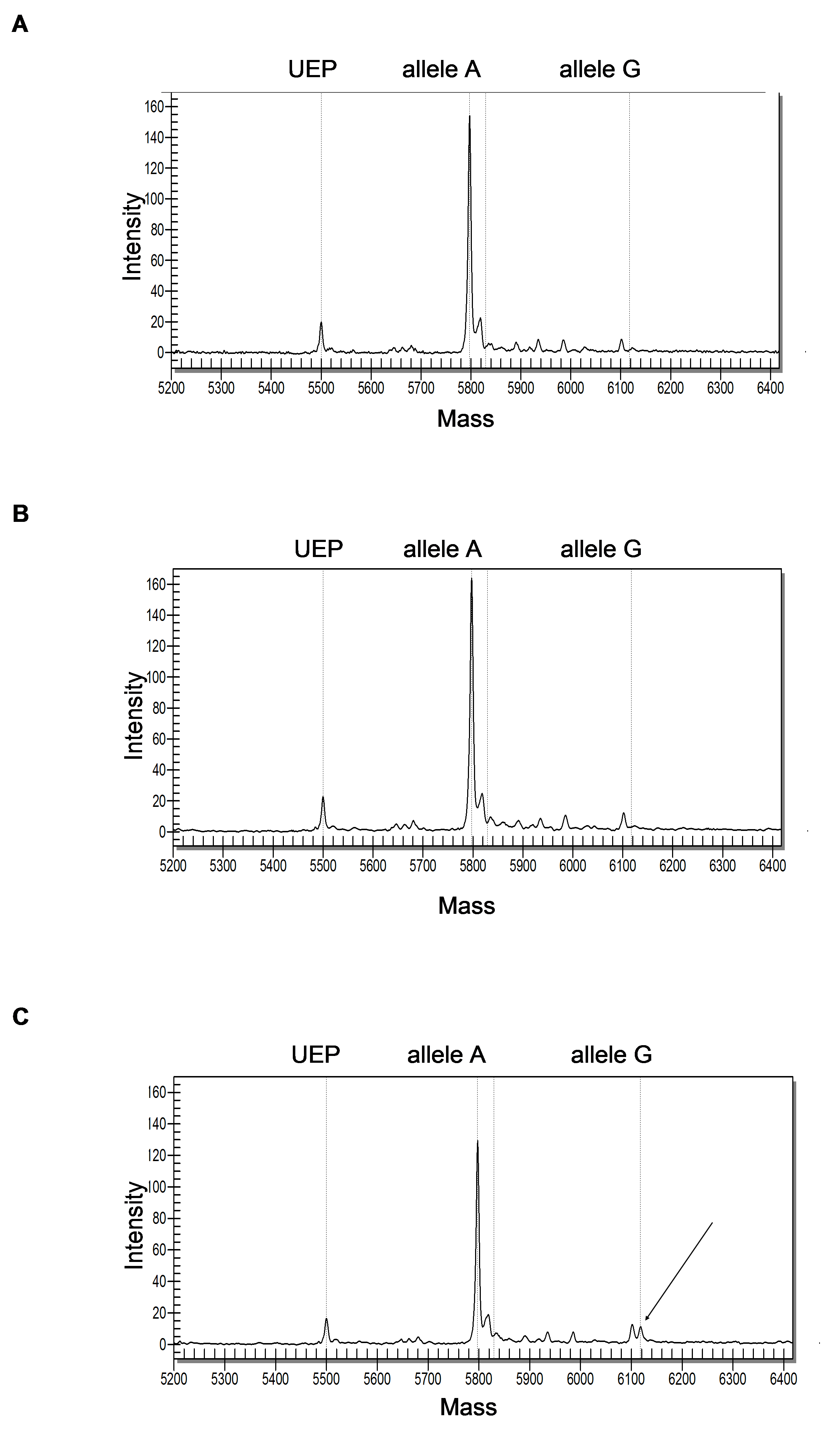

Supplement: Figure S4 — Mass spectra showing PLAC4 RNA-SNP genotypes of placenta, third trimester maternal plasma and whole blood samples. (A) a homozygous placental RNA sample showing a single peak A with unextended primers (UEP). (B) third trimester maternal plasma RNA showing a single peak A as the placental RNA sample. (C) third trimester maternal whole blood RNA showing a peak A and a minor peak G as indicated by the arrow. (1.09 MB TIF) [file pone.0005858.s004.tif]

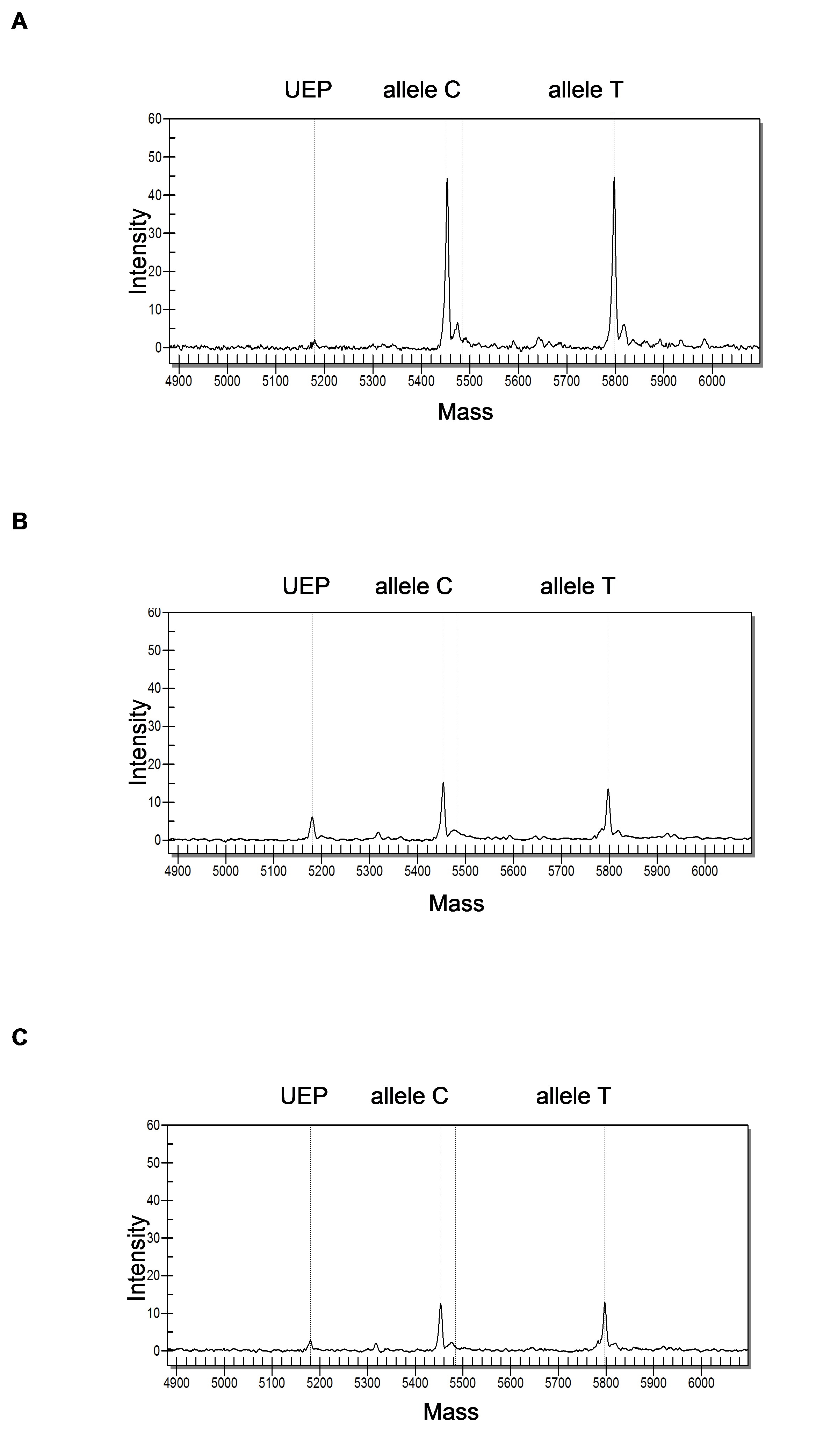

Supplement: Figure S5 — Mass spectra showing CSHL1 RNA-SNP genotypes of placenta, third trimester maternal plasma and whole blood samples. (A) a heterozygous placental RNA sample showing 2 peaks, allele C and allele T with unextended primers (UEP). (B) third trimester maternal plasma RNA also showing 2 peaks as the placental RNA sample. (C) third trimester maternal whole blood RNA showing both peaks of allele C and allele T. (1.07 MB TIF) [file pone.0005858.s005.tif]
