# Supplementary material for: Placenta-Derived Fetal Specific mRNA Is More Readily Detectable in Maternal Plasma than in Whole Blood
Source: PLoS One. 2009 Jun 10;4(6):e5858. doi: 10.1371/journal.pone.0005858 (PMC2690655; doi:10.1371/journal.pone.0005858)
Supplement: Table S1 — Primer, probe and calibrator sequences and reaction conditions for quantitative real-time RT-PCR assays. (0.08 MB DOC) [file pone.0005858.s006.doc]

**Online Supporting Information**

Table S1

Primer, probe and calibrator sequences and reaction conditions for quantitative real-time RT-PCR assays

|  |  |  | **Annealing** | **Final conc.** |
| --- | --- | --- | --- | --- |
| **Transcript** | **Sequence** |  | **Temp (^o^C)** | **for RT-PCR (nM)** |
| *CSH1* | F primer | 5'- CATGACTCCCAGACCTCCTTC -3' | 56 | 300 |
|  | R primer | 5'- TGCGGAGCAGCTCTAGATTG -3' |  | 300 |
|  | Probe | 5'-(FAM) TTCTGTTGCGTTTCCTCCATGTTGG (TAMRA)-3' |  | 100 |
|  | Standard | 5'- TGCGGAGCAGCTCTAGATTGGATTTCTGTTGCGTTTCCTCCATGTTGGAGGG-  TGTCGGAATAGAGTCTGAGAAGCAGAAGGAGGTCTGGGAGTCATGC -3' |  |  |
| *KISS1* | F primer | 5'- GCCCAGGCCAGGACTGA -3' | 57 | 300 |
|  | R primer | 5'- GCCAAGAAACCAGTGAGTTCATC -3' |  | 300 |
|  | Probe | 5'-(FAM) CCTCAAGGCACTTCTAGGACCTGGCTCTTC (TAMRA)-3' |  | 100 |
|  | Standard | 5'- CTGCCCAGGCCAGGACTGAGGCAAGCCTCAAGGCACTTCTAGGACCTGGCT-  CTTCTCACCAAGATGAACTCACTGGTTTCTTGGCAG -3' | |  |
| *PLAC4* | F primer | 5'- CCTTTCCCCCTTATCCAACT -3' | 60 | 400 |
|  | R primer | 5'- GTACTGGTTGGGCTCATTTTCT -3' |  | 400 |
|  | Probe | 5'-(FAM) CCCTAGCCTATACCC (MGBNFQ)-3' |  | 100 |
|  | Standard | 5'- CACCTTTCCCCCTTATCCAACTAGCCCTAGCCTATACCCTCTGCTGCCCAAG-  AAAATGAGCCCAACCAGTACAC -3' | | |
| *PLAC1* | F primer | 5'- ATTATCCCCAGCTGCCAGAA -3' | 56 | 400 |
|  | R primer | 5'- GCAGCCAATCAGATAATGAACCA -3' |  | 400 |
|  | Probe | 5'-(FAM) AAGAAATCCTCACTGGACGGCTTCCTG (TAMRA)-3' |  | 200 |
|  | Standard | 5'- ACAAATTATCCCCAGCTGCCAGAAGAAGAAATCCTCACTGGACGGCTTCCTG-  TTTCCTGTGGTTCATTATCTGATTGGCTGCAGG -3' | | |
| *DEFA4* | F primer | 5'- TCTATTTCCTTTGCATGGGATAAAA -3' | 56 | 300 |
|  | R primer | 5'- GCAGACCATGCCCCTTG -3' |  | 300 |
|  | Probe | 5'-(FAM) CTTCAGGTTTCAGGCTCA (MGBNFQ)-3' |  | 100 |
|  | Standard | 5'- TATCTATTTCCTTTGCATGGGATAAAAGCTCTGCTCTTCAGGTTTCAGGCTCA-  ACAAGGGGCATGGTCTGCTC -3' | | |
| *CEACAM8* | F primer | 5'- ATCTTATGAGTGAAGAAGTAACTGGC -3' | 58 | 400 |
|  | R primer | 5'- TTGTTGCTGGAGATGGAGG -3' |  | 400 |
|  | Probe | 5'-(FAM) CGTACATCCGGAGACT (MGBNFQ)-3' |  | 100 |
|  | Standard | 5'- TAAATCTTATGAGTGAAGAAGTAACTGGCCAGTTCAGCGTACATCCGGAGAC-  TCCCAAGCCCTCCATCTCCAGCAACAACTC -3' | | |
| *OLFM4* | F primer | 5'- GCTCCAGCCGCAGCTTAG -3' | 60 | 300 |
|  | R primer | 5'- GGAGCCGGTGAAATTGGAA -3' |  | 300 |
|  | Probe | 5'-(FAM) AGGTTCTGTGTCCCAGTTG (MGBNFQ)-3' |  | 100 |
|  | Standard | 5'- CCAGCTCCAGCCGCAGCTTAGGCAGCGGAGGTTCTGTGTCCCAGTTGTTTT-  CCAATTTCACCGGCTCCGTG -3' | | |
| *FLCN* | F primer | 5'- TGCAGCTAAGCAGCCAACTG -3' | 60 | 200 |
|  | R primer | 5'- CATTCATGGTGCCTTGGAGACT -3' |  | 200 |
|  | Probe | 5'-(FAM) AACGTCAGGCCTGTTG (MGBNFQ)-3' |  | 100 |
|  | Standard | 5'- CGTGCAGCTAAGCAGCCAACTGCAGAAACGTCAGGCCTGTTGCAGTCTCCA-  AGGCACCATGAATGCC -3' | | |
| *ORM1* | F primer | 5'- TTGCGCATTCCCAAGTCA -3' | 60 | 300 |
|  | R primer | 5'- CAGTGGCTCACACTTATCCTTTTTC -3' |  | 300 |
|  | Probe | 5'-(FAM) TGTCGTGTACACCGATT (MGBNFQ)-3' |  | 100 |
|  | Standard | 5'- GCTTGCGCATTCCCAAGTCAGATGTCGTGTACACCGATTGGAAAAAGGATAA-  GTGTGAGCCACTGGAG -3' | | |
| *MMP8* | F primer | 5'- AGGCAGATATCAACATTGCTTTTTAC -3' | 60 | 600 |
|  | R primer | 5'- GATTCCATTGGGTCCATCAAA -3' |  | 600 |
|  | Probe | 5'-(FAM) AAAGAGATCACGGTGACAAT (MGBNFQ)-3' |  | 150 |
|  | Standard | 5'- GAGAGGCAGATATCAACATTGCTTTTTACCAAAGAGATCACGGTGACAATTCT-  CCATTTGATGGACCCAATGGAATCCTT -3' | | 150 |
| *MPO* | F primer | 5'- CCACCAAAACCGATCACCAT -3' | 56 | 300 |
|  | R primer | 5'- CACTCCTCGCCTGCATCAT -3' |  | 300 |
|  | Probe | 5'-(FAM) CTTCCTGAACTGGGTACC (MGBNFQ)-3' |  | 100 |
|  | Standard | 5'- TCCCACCAAAACCGATCACCATCCCGGAGCTTCCTGAACTGGGTACCGATGA-  TGCAGGCGAGGAGTGGG -3' |  |  |
|  |  |  |  |  |
| FAM, 6-carboxyfluorescein | |  |  |  |
| MGNNFQ, minor groove binding non-fluorescent quencher | | |  |  |
