# Supplementary material for: Placenta-Derived Fetal Specific mRNA Is More Readily Detectable in Maternal Plasma than in Whole Blood
Source: PLoS One. 2009 Jun 10;4(6):e5858. doi: 10.1371/journal.pone.0005858 (PMC2690655; doi:10.1371/journal.pone.0005858)
Supplement: Table S2 — Primer sequences for reverse transcription and PCR amplification of the PLAC4 and CSHL1 SNP. (0.03 MB DOC) [file pone.0005858.s007.doc]

**Online Supporting Information**

Table S2

Primer sequences for reverse transcription and PCR amplification of the *PLAC4* and *CSHL1* SNP

|  |  |  |
| --- | --- | --- |
| **Gene** | **Primer** | **Sequence** |
| ***PLAC4*** | Gene-specific primer for reverse transcription | 5' -GTATATAGAACCATGTTTAGGCCAG-3' |
| **(rs8130833)** | Forward PCR primer | 5' -**ACGTTGGATG**GTATTGCAACACCATTTGGG-3' |
|  | Reverse PCR primer | 5' -**ACGTTGGATG**TAGAACCATGTTTAGGCCAG-3' |
| ***CSHL1*** | Gene-specific primer for reverse transcription | 5' -GCACTGGGGAGGGGTCA-3' |
| **(rs2246207)** | Forward PCR primer | 5' -**ACGTT**AGAAATCCAACTTAGAGCTGCTCCA-3' |
|  | Reverse PCR primer | 5' -**ACGTTGGATG**GAGGGTCTGCCCAGTCAGGT-3' |
|  |  |  |
| Bold fonts indicate the tag. The tags on the 5' ends of each PCR primers increase the masses of unused PCR | | |
| primers so it would fall outside the analytical mass range in the MALDI-TOF analysis. | | |
|  | |  |
