# Supplementary material for: Placenta-Derived Fetal Specific mRNA Is More Readily Detectable in Maternal Plasma than in Whole Blood
Source: PLoS One. 2009 Jun 10;4(6):e5858. doi: 10.1371/journal.pone.0005858 (PMC2690655; doi:10.1371/journal.pone.0005858)
Supplement: Table S3 — Sequences and molecular weights of the extension primer and the expected extension products for each of the alleles of the PLAC4 and CSHL1 SNPs. (0.03 MB DOC) [file pone.0005858.s008.doc]

**Online Supporting Information**

Table S3

Sequences and molecular weights of the extension primer and the expected extension products for each of the alleles of the *PLAC4* and *CSHL1* SNPs

|  |  |  |  |
| --- | --- | --- | --- |
| **Gene** | **Expected peak** | **Sequence (5'-3')** | **Molecular weight (Da)** |
| ***PLAC4*** | Unextended primer | AGGCCAGATATATTCGTC | 5498.6 |
| **(rs8130833)** | Extension product for allele A | AGGCCAGATATATTCGTC**A** | 5795.8 |
|  | Extension product for allele G | AGGCCAGATATATTCGTC**GT** | 6116.0 |
| ***CSHL1*** | Unextended primer | TATGACACCTCGGACAG | 5179.4 |
| **(rs2246207)** | Extension product for allele C | TATGACACCTCGGACAG**C** | 5452.6 |
|  | Extension product for allele T | TATGACACCTCGGACAG**TG** | 5796.8 |
|  |  |  |  |
| Bold fonts indicate the extended dNTPs and ddNTPs. | | |  |
